# Automated workflow composition in mass spectrometry-based proteomics

**DOI:** 10.1093/bioinformatics/bty646

**Published:** 2018-07-24

**Authors:** Magnus Palmblad, Anna-Lena Lamprecht, Jon Ison, Veit Schwämmle

**Affiliations:** 1 Center for Proteomics and Metabolomics, Leiden University Medical Center, RC Leiden, The Netherlands; 2 Department of Information and Computing Sciences, Utrecht University, CC Utrecht, The Netherlands; 3 National Life Science Supercomputing Center, Technical University of Denmark, Kongens Lyngby, Denmark; 4 Department of Biochemistry and Molecular Biology and VILLUM Center for Bioanalytical Sciences, University of Southern Denmark, Odense, Denmark

## Abstract

**Motivation:**

Numerous software utilities operating on mass spectrometry (MS) data are described in the literature and provide specific operations as building blocks for the assembly of on-purpose workflows. Working out which tools and combinations are applicable or optimal in practice is often hard. Thus researchers face difficulties in selecting practical and effective data analysis pipelines for a specific experimental design.

**Results:**

We provide a toolkit to support researchers in identifying, comparing and benchmarking multiple workflows from individual bioinformatics tools. Automated workflow composition is enabled by the tools’ semantic annotation in terms of the EDAM ontology. To demonstrate the practical use of our framework, we created and evaluated a number of logically and semantically equivalent workflows for four use cases representing frequent tasks in MS-based proteomics. Indeed we found that the results computed by the workflows could vary considerably, emphasizing the benefits of a framework that facilitates their systematic exploration.

**Availability and implementation:**

The project files and workflows are available from https://github.com/bio-tools/biotoolsCompose/tree/master/Automatic-Workflow-Composition.

**Supplementary information:**

Supplementary data are available at *Bioinformatics* online.

## 1 Introduction

Biological research today routinely involves the application of multiple, diverse computational methods in a sequence of operations to convert raw measurements into condensed results for biological interpretation. In the provision of these methods as application software, we discern two opposing paradigms. First, integrated software packages provide the scientist with a convenient one-stop-shop, with user-friendly but often limited functionality that usually is operated through a graphical user interface. For example, CompOmics (biotools:compomics-utilities) (Barsnes *et al.*, 2011), MaxQuant/Perseus (biotools:maxquant) (Cox and Mann, 2008), Skyline (biotools:skyline) (MacLean *et al.*, 2010) and Scaffold (Searle, 2010) are especially suited for the fine-grained analysis of a single experiment, dataset or spectrum. The contrasting paradigm encapsulates one or a few closely related methods into discrete, stand-alone tools, enabling the expert user with a powerful command-line interface. Such tools excel as remixable components in automatic data analysis pipelines for high-throughput processing, often referred to as *workflows*. The construction of bespoke workflows such as InSilicoSpectro (biotools:insilicospectro) (Colinge *et al.*, 2006), OpenMS (biotools:openms) (Röst *et al.*, 2016) and Proteomatic (biotools:proteomatic) (Specht *et al.*, 2011), affords the scientist with valuable freedom and flexibility. However, in an era of proliferating analytical tools (Duck *et al.*, 2016), a multitude of data formats, high turnover of functionalities, and sometimes scant documentation, the construction and maintenance of such pipelines must address significant challenges:

1. *Discovery* of all potentially applicable tools for the task at hand;
2. *Annotation* of tools to understand their specific function(s) and the technical requirements for connecting other tools, in particular supported input and output data formats;
3. *Composition* of workflows from the available tools including dependencies such as format converters and other utilities;
4. *Implementation* of workflows by developing their logic (e.g. as scripts) and their parameters;
5. *Management* of data processing by deploying, executing and monitoring workflows;
6. *Maintenance* of workflows by updating tools to new versions, or substituting obsolete or broken workflow components.
7. *Validation* of created workflows by checking tool interoperability, their usability and furthermore benchmarking of the resulting data.

Moreover *reproducibility* is a major concern. When investigating the effect of a candidate drug in a disease model, researchers use inbred, genetically near-identical, animals to isolate the effect of the drug from a background of biological variability. Conversely, toxicologists use outbred animals to cover as much genetic variability as possible and make sure a negative result is not specific to a particular genotype. Analogously, we may want to investigate the effect of a particular tool or operation, ceteris paribus, and therefore generate workflows that are similar except for the tool or operation under investigation. On the other hand, we are often interested in validating *results*. For this purpose, we generate semantically equivalent workflows having a minimum number of shared tools, to verify the biological results are not tool-dependent. Here we are concerned with computational reproducibility—can another researcher arrive at the same results and conclusions starting from the same data? As data formats become obsolete, software ceases to be updated or disappears entirely, most analyses will not be reproducible indefinitely. If the analysis relies on a single software package, the entire analysis depends on the availability, and continued development, of that package. Switching to another package (if at all available) will almost certainly produce different results.

In general, these challenges are overcome by often manual efforts including literature mining and testing of software tools, their interoperability and the results. To assist the beleaguered bioinformatician, various workflow management systems, such as Taverna (biotools:taverna) (Wolstencroft *et al.*, 2013), Galaxy (biotools:galaxy) (Afgan *et al.*, 2016), KNIME (biotools:knime) (Berthold *et al.*, 2009), Triana (Taylor *et al.*, 2007) and Kepler (biotools:kepler) (Ludäscher *et al.*, 2006) to name a few [see also recent reviews (Leipzig, 2017; Möller *et al.*, 2017) and Galaxy examples (Boekel *et al.*, 2015; Jagtap *et al.*, 2014; Sheynkman *et al.*, 2014)], ease the development and execution of workflow logic. The frequently used graphical workflow models abstract from the syntactical details of classical programming languages, simplifying workflow creation and execution. Although any workflow in principle can be created in a workflow management system, solutions to specific data analysis problems often do not exist, requiring customisation of the environment with new tools. In such cases, comprehensive and consistent information about bioinformatics software enables a researcher to discover (find, understand, compare and select) tools during workflow composition. Within the ELIXIR Tools and Data Services Registry (Ison *et al.*, 2015; https://bio.tools) tools are described in terms from the EDAM ontology (Ison *et al.*, 2013), which provides a controlled vocabulary for bioinformatics concepts, including data types, formats and identifiers, operations and common topics. EDAM includes for each concept a persistent and unique identifier, a preferred term, definition and (optionally) one or more synonyms. It thus enables a precise and practical description of the scientific function of a tool, helping the investigation of tool interoperability, which is essential for the composition and evaluation of workflows from the entire space of possible tool combinations. Tools with matching input/output type and file format are likely to serve as compatible components of a workflow given a set of correct and sufficiently dense tool annotations, but still need to be tested as workflow compositions; even tools which are fully compatible in principle do not necessarily provide operational workflows and correct data treatment.

From these observations the wish to compose and test the applicability of entire workflows automatically seems to be a natural next step, and indeed several works exist that have tried to achieve this in the bioinformatics domain. Examples include the semantic service composition approaches in myGrid (Chen *et al.*, 2003; Lord *et al.*, 2004), different agent-based approaches as reviewed in (Merelli *et al.*, 2007), the meanwhile discontinued BioMOBY registry (DiBernardo *et al.*, 2008), the OWL-based SADI framework with its SHARE client for web service pipelining (Wilkinson *et al.*, 2010), the template-based semantic workflow descriptions by which Wings extends the Pegasus workflow system (Gil *et al.*, 2004, 2007), the ASKALON workflow framework with its abstract workflow description language (Qin and Fahringer, 2012) and the PROPHETS framework that makes use of temporal-logic synthesis (Lamprecht, 2013; Lamprecht *et al.*, 2009, 2010; Naujokat *et al.*, 2012). In the general computer science community, the topic of automated synthesis and planning of workflows has been discussed at least since the 1990s (Margaria and Steffen, 1998; Moreno and Kearney, 2002; Steffen *et al.*, 1993), and gained new attention in the early 2000s when (semantic) web services became popular (Aggarwal *et al.*, 2004; Rao and Su, 2005). All these approaches have in common that their practical success depends on rich, consistent component annotation, but this information is usually not available. The study described in Lamprecht *et al.* (2011) used PROPHETS to demonstrate on the EMBOSS sequence analysis tool suite, which was the first collection of tools completely annotated using the EDAM ontology, how a dense and systematic annotation boosts what automatic workflow composition techniques can facilitate. It did however not compare and evaluate implementations of the different suggested workflows against each other.

In this article, we add this next logical step. We explore the value of formalized semantic tool descriptions for guided construction of practical workflows for mass spectrometry (MS)-based proteomics. Workflow creation follows a minimal framework, i.e. definition of operations, input files and output files of the complete workflow. We use PROPHETS to synthesize workflows based on a library of EDAM-annotated tools registered within bio.tools. We implement different workflows for four typical use cases in the analysis of MS data: peptide retention time prediction, protein identification and enrichment analysis, localization of phosphorylation and protein quantitation using isotopic labeling. Finally, we test and compare the workflows on public data and thereby demonstrate both the feasibility and potential of automatic workflow composition in bioinformatics exemplified for MS-based proteomics.

## 2 Materials and methods

**Fig. 1.**
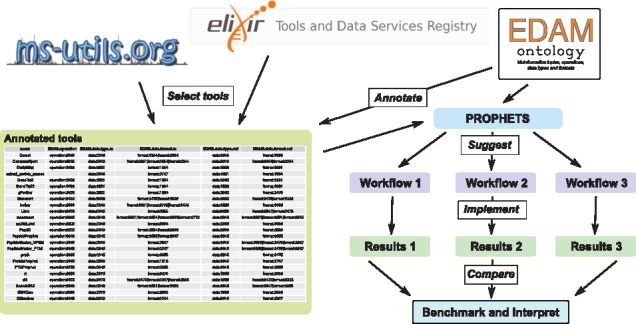
Schematic outline of the workflow composition. PROPHETS suggests workflows for a selection of tools from ms-utils.org and bio.tools, annotated in terms from the EDAM ontology, under constraints such as requested operations or input and output formats. The resulting workflows are implemented and tested on public data

To illustrate how tool annotation and automated workflow creation can help to analyze real data, we consider an exemplary set of tools and four use cases of increasing complexity, that are described (below) in EDAM terms (version 1.18). Multiple executable workflows are composed from a list of annotated tools prevalent in proteomics data analysis (Fig. 1). In the following, EDAM terms are underlined and linked to the official representation, e.g. post-translational modification (PTM) identification, or given by its ID in brackets, [operation:3645].

### 2.1 Proteomics use cases

**Use case no. 1.** Suppose a chemist has developed a new chromatographic column and wants to know how the chromatographic separation of the peptides depends on their amino acid composition (Meek, 1980). Using the column in a liquid chromatography tandem MS analysis of an. *Escherichia coli* tryptic digest yields multiple MS spectra in the instrument vendor’s Thermo RAW format. The chemist would like to get the Amino acid index (hydropathy), defined as ‘hydrophobic, hydrophilic or charge properties of amino acids’ for each of the 20 amino acids (generally, an Amino acid index is ‘a table of 20 numerical values which quantify a property, e.g. physicochemical or biochemical, of the common amino acids’). The chemist knows that at least one peptide identification operation with validation of peptide-spectrum matches and one retention time prediction operation will be necessary to get the intended index, and that protein identification is not needed (our chemist has no interest in the proteins the peptides were derived from, indeed, they would have been fine with synthetic peptides).

**Use case no. 2.** Next, our scientist faces the common task of protein identification and interpretation of lists of identified proteins by enrichment analysis, and again starts from MS spectra in the Thermo RAW format. After peptide database search and protein identification, the list of proteins identified via UniProt accessions should be analysed by gene-set enrichment analysis with respect to KEGG pathways and annotations, reporting KEGG pathway IDs and associated *p*- or *q*-values.

**Use case no. 3.** The identification and localization of post-translational modifications is another frequent task in MS-based proteomics. Suppose our scientist is conducting a phosphoproteomic study and now wants to identify the phosphopeptides and localize the phosphorylations within the peptides, that is, a PTM identification after or concurrent with the peptide database search. To have some control of the false discovery rate (FDR) of both the peptide identifications and phosphorylation localization, a PTM localization and validation of peptide-spectrum matches are necessary.

**Use case no. 4.** Our scientist has now graduated to more challenging problem of protein quantitation. Additionally to the operations peptide database search and validation of peptide-spectrum matches, a quantification step is needed, specifically using iTRAQ.

### 2.2 Proteomics tools

As evident from the use case descriptions above, data analysis in MS-based proteomics often involves many separate tasks, such as raw data transformation, calibration, feature extraction, peptide identification, protein inference, quantitation and higher-level biological analyses. For each of these tasks a number of software tools are available, some commercial, many free, in addition to larger integrated environments that can perform many of these tasks sequentially. In preparation for this study, the majority of tools listed on ms-utils.org were manually annotated and registered in bio.tools. The EDAM annotations for selected tools were subsequently retrieved from bio.tools when creating the workflow specifications (Supplementary Table S1).

### 2.3 Automatic workflow composition

Automatic generation of computer programs from abstract specifications (also referred to as synthesis) has been studied for decades (Bodik and Jobstmann, 2013). We use the PROPHETS (Process Realization and Optimization Platform using Human-readable Expression of Temporal-logic Synthesis) framework (Version 1.3) for automatic workflow composition. It is based on a synthesis method that uses the Semantic Linear Time Logic (SLTL) for workflow specification and thus allows for a precise tailoring of the specification to the userse intents via SLTL constraints (Steffen *et al.*, 1993). Working with PROPHETS comprises essentially three phases: Domain Modeling, Workflow Specification, and Workflow Synthesis.

#### 2.3.1 Domain modeling

Tools must be annotated with input and output data types to be considered. These types, and also the tool names, must be classified in taxonomies that define categories of related types and tools, and thus provide a controlled vocabulary for the annotations. The annotations and the taxonomies together with a (possibly empty) set of SLTL constraints (for defining general properties that the synthesized workflows have to fulfill) constitute the *domain model* for PROPHETS.

**Fig. 2.**
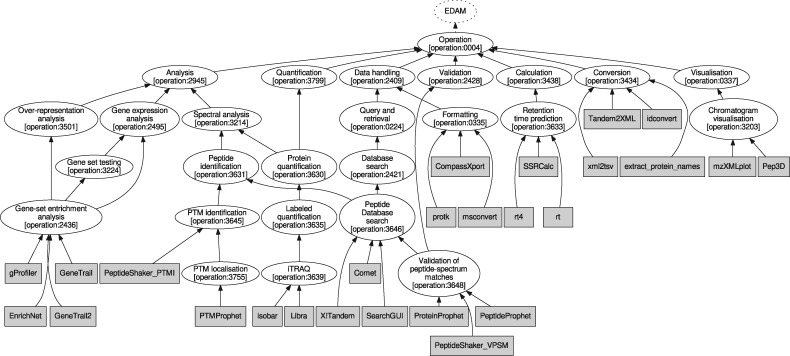
Tool taxonomy, consisting of the tools in the domain model and the corresponding parts of the EDAM Operation sub-ontology

The domain model for the use cases was generated from the tool annotations (indicated in Fig. 2 and comprehensively defined by Supplementary Table S1). Every tool is listed with its name, an EDAM Operation, and for each of input and output, an EDAM Data and one or more EDAM Format terms. For reasons of simplicity we limited the annotations to one input/output per tool in this study, although the synthesis framework is generally able to handle multiple inputs and outputs per tool.

The domain model comprises a tool taxonomy of tool names and the corresponding parts of the EDAM operation subontology (Fig. 2), and a type taxonomy consisting of parts of the EDAM Data and Format subontologies (Supplementary Fig. S1). These taxonomies make it possible to refer to groups (classes) of tools, data types and formats in the workflow specifications, rather than concrete instances. The only domain constraints that we defined for this study are to avoid using msconvert (biotools:msconvert) and idconvert (biotools:idconvert) twice directly after itself, in order to prevent pointless chains of format conversions.

#### 2.3.2 Workflow specification

Workflow specifications in PROPHETS comprise the input data type(s), the output data types(s), and possible additional constraints that the synthesized sequence must fulfill. Also without knowledge of SLTL, the constraints can easily be formulated, as PROPHETS includes a Constraint Editor that provides natural-language templates for common workflow constraints and ensures that the gaps in the cloze texts can only be filled with terms from the domain model. This flexible way of working with constraints allows for a very fine-grained and yet intuitive tailoring of the workflow specification to the intentions of the workflow developer. The workflow specifications for the four use cases are shown in the Results section. Together with the domain model, the workflow specification is the input for the synthesis algorithm.

#### 2.3.3 Workflow synthesis

PROPHETS has various parameters for the execution of the synthesis algorithm (cf. the framework’s online manual). For our four use cases, PROPHETS was configured to use the pipelining synthesis process, the simple goal constraint, the ‘tsmyoo’ synthesis algorithm and a bounded search strategy. Generally we let the search only run until the first lengths for which solutions were found, as to only take into account the shortest solutions. Typically more solutions are found for greater lengths, but they often just comprise additional steps, which are not required to meet the specification and can thus be omitted.

### 2.4 Workflow implementation and evaluation

Tool compatibility from matching data and file formats does not automatically guarantee interoperability. Experience tells us that subtle differences in the *interpretations* of data format schemata rule out some—perhaps the majority—of the proposed workflows. It is therefore essential to validate the automatically created workflows on real input data. PROPHETS is able to create executable workflow models directly from the synthesized sequences. In this study, however, the basic tool descriptions are insufficient to configure all the tool parameters automatically. The synthesized workflows are thus abstract representations of analysis pipelines rather than executable workflow instances. We manually created instances of selected synthesis solutions (proposed workflows) in order to validate and execute them. They were implemented as Shell scripts, so that already available scripts and workflow fragments could easily be reused. We created a static environment by making the used software available in a docker container on github.com/bio-tools/biotoolsCompose/tree/master/Automatic-Workflow-Composition. The container contains the scripts to rerun the different workflows or adapt them to other datasets.

## 3 Results

We describe the workflow specifications derived from the use-case descriptions, and the generic workflows that PROPHETS proposed given the specifications (summarized in Table 1). We discuss and compare the data output from implementing selected workflows and running them on the example datasets referred to in the use case narratives.

**Table 1.** Summary of workflow specifications

Use case	Workflow input	Workflow output	Workflow constraints
No. 1	Mass spectr. spectra in Thermo RAW	Amino acid index (hydropathy) in any format	(i) Use peptide identification; (ii) Use validation of peptide-spectrum matches; (iii) Use retention time prediction; (iv) Do not use protein identification
No. 2	Mass spectr. spectra in Thermo RAW	Pathway or network in any format	(i) Use peptide identification; (ii) Use gene-set enrichment analysis; (iii) Use gene-set enrichment analysis only after peptide identification; (iv) Use ProteinProphet only after PeptideProphet.
No. 3	Mass spectr. spectra in Thermo RAW	Protein identification in any format	(i) Use PTM identification; (ii) Use PTM identification only after Validation of peptide-spectrum matches; (iii) Use validation of peptide-spectrum matches only after peptide database search; (iv) Do not use validation of peptide-spectrum matches more than once.
No. 4	Mass spectr. Spectra in Thermo RAW	Gene expression profile in any format	(i) Use iTRAQ; (ii) Use iTRAQ only after validation of peptide-spectrum matches

### 3.1 Synthesized workflows

**Use case no. 1.** translates into a specification with MS spectra [data: 0943] in Thermo RAW [format:3712] as workflow input and Amino acid index (hydropathy) [data:1506] as workflow output. Straightforward from the natural-language workflow description of in Section 2.1, constraints are used to enforce the use a peptide identification [operation:3631] operation, a validation of peptide-spectrum matches [operation:3648] operation, and a retention time prediction [operation:3633] operation, and to avoid the use of protein identification [operation:3767].

**Fig. 3.**
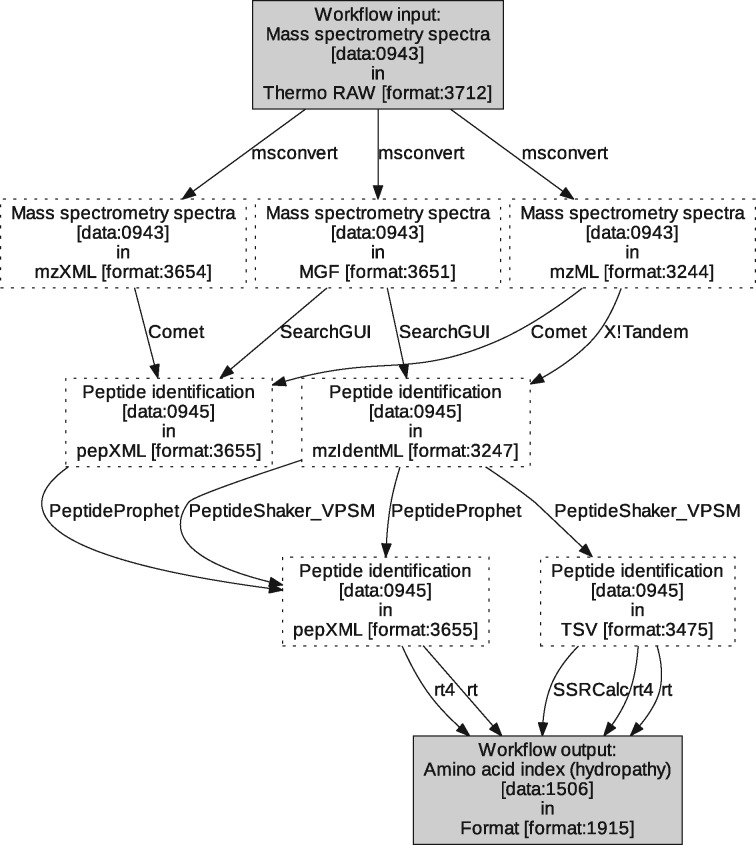
Automata-like representation of the set of proposed solutions (synthesized workflows of Length 4) for use case no. 1. The graph nodes represent data items (described by EDAM Data and Format terms), and the directed edges are labeled with tools from the domain model, denoting that the tool can be used with the inputs and outputs of the edge source and target, respectively. Each path through this graph from the workflow input to output node is a workflow that fulfills the specification given to PROPHETS

The minimal workflow that PROPHETS finds for this specification comprises four steps: peptide identification [operation:3631], validation of peptide-spectrum matches [operation:3648] and retention times prediction [operation:3633], preceded by a formatting step to convert the MS spectra [data:0943] in Thermo RAW [format:3712] into a format compatible with the peptide identification tools. In total PROPHETS finds 31 possible workflows of Length 4 that fulfill the specification. They are summarized in Figure 3. If we let the synthesis look a bit further and return solutions up to a length of 5, several more workflows are possible (388 in total, see also Supplementary Fig. S2). Looking up to a length of 6, there are even 3, 127 possible workflows for this specification (see Supplementary Fig. S3). From these workflows, we selected four for implementation and evaluation:

- msconvert → Comet → PeptideProphet → rt4
- msconvert → Comet → PeptideProphet → xml2tsv → SSRCalc
- msconvert → X! Tandem → Tandem2XML → PeptideProphet → rt4
- msconvert → X! Tandem → Tandem2XML → PeptideProphet → xml2tsv → SSRCalc

**Use case no. 2** implies a specification with MS spectra [data: 0943] in Thermo RAW [format:3712] as workflow input and pathway or network [data:2600] as workflow output. Constraints are used to express that peptide identification [operation:3631] and gene-set enrichment analysis [operation:2436] should be used, and in this order, and that ProteinProphet should only be used after PeptideProphet. At least six services are required in this case to fulfill the specification (see also Supplementary Fig. S4). We implemented three of the 20 proposed workflows of Length 6 for evaluation:

- msconvert → Comet → PeptideProphet → ProteinProphet → extract_protein_names → GeneTrail2
- msconvert → Comet → PeptideProphet → ProteinProphet → extract_protein_names → EnrichNet
- msconvert → Comet → PeptideProphet → ProteinProphet → extract_protein_names → gProfileR

**Use case no. 3** suggests a specification that again has MS spectra [data:0943] in Thermo RAW [format:3712] as workflow input, and protein identification [data:0945] as workflow output. Constraints are added to formulate that PTM identification [operation:3645] is required, that PTM identification [operation:3645] depends on validation of peptide-spectrum matches [operation:3648], and that validation of peptide-spectrum matches [operation:3648] depends on peptide database search [operation:3646]. Furthermore, a constraint ensures that Validation of peptide-spectrum matches [operation: 3648] is not used more than once. There are 13 workflows of Length 4 suggested by PROPHETS in this case (see also the graph in Supplementary Fig. S5D). We selected two of them to implement and evaluate:

- msconvert → Comet → PeptideProphet → PTMProphet
- msconvert → SearchGUI → PeptideShaker_VPSM → Peptide-Shaker_PTMI

For **use case no. 4**, the workflow input is once again MS spectra [data:0943] in Thermo RAW [format:3712], and the desired workflow output is Gene expression profile [data:0928] in any format [format:1915]. Constraints are used to express that a quantification [operation:3630] step, specifically iTRAQ [operation: 3639] is required, and that iTRAQ [operation:3639] depends on validation of peptide-spectrum matches [operation:3648]. Again, the shortest solutions that PROPHETS finds have a length of four. The set of solutions comprises 16 possible workflows (see also Supplementary Fig. S6). We implemented two of them for evaluation:

- msconvert → SearchGUI → PeptideShaker_VPSM → isobar
- msconvert → Comet → PeptideProphet → Libra

### 3.2 Implementation and results of selected workflows

**Use case no. 1.** As test data, we used the same *E.coli* digest reversed-phase chromatography—amaZon ion trap [MS:1001542] dataset previously used to optimize peptide identification (Holl *et al.*, 2015), available on cpm.lumc.nl/export/public_datasets. The tandem mass spectra were searched against the UniProt reference proteome for *E. coli* (strain K12), proteome up000000318 downloaded 20160617 (4,254 sequences). A mass measurement error tolerance of 0.5 Da was used, allowing for isotope error (resulting in a search window [−0.5, 3.5] Da with Comet or [−0.5, 2.5] with X! Tandem). Methionine oxidation was considered as a variable modification and cysteine carbamidomethylation as the only fixed modification use cases 1 and 2.

**Fig. 4.**
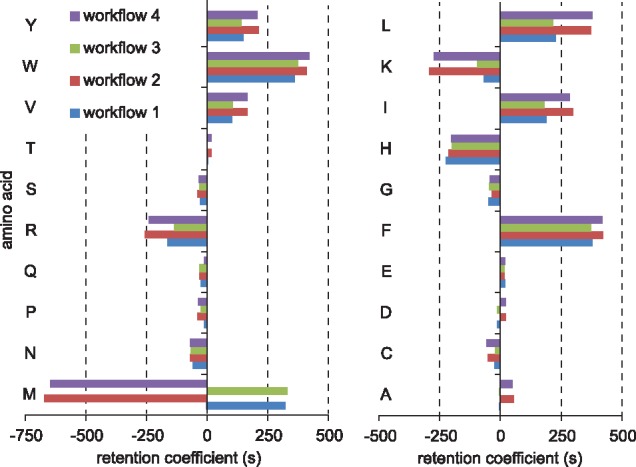
Comparison of retention time coefficients in use case no. 1

The four workflows produced similar retention time models (Fig. 4). The retention coefficients are similar for most amino acids. The retention time predictor has a larger influence on the variety of the results than the search engine. The most salient exceptions are methionine (the residue with variable modification) and the basic residues lysine and arginine which are usually only found in the C-terminus of tryptic peptides. SSRCalc treats termini separately whereas rt4 does not.

**Use case no. 2.** Three workflows, all based on Comet but using the three different enrichment tools EnrichNet (Glaab *et al.*, 2012), GeneTrail2 (Stöckel *et al.*, 2016) and g:Profiler (Reimand *et al.*, 2016), suggested by PROPHETS were realized. Here we show three using the Comet peptide database search tool (Fig. 3). EnrichNet and GeneTrail2 are Web Services that require some additional specification in the workflow to set up, call and retrieve data from the servers. These embedded workflow components encapsulate the tools and can be reused in other workflows integrating these services. g:Profiler can be installed locally and accessed running on a local instance of R.

To test the workflows, we used all eight LTQ Orbitrap Velos [MS:1001742] gas-phase fractionation datasets from a characterization of the platelet granule proteome (Zufferey *et al.*, 2014), ProteomeXchange dataset PXD000618. The data were searched against the UniProt reference proteome for *Homo sapiens*, proteome up000005640 downloaded 20160617 (70 615 sequences). Mass measurement error up to 5 ppm was allowed, with isotope error as before. Only peptides with PeptideProphet probability of at least 0.8 and 7 residues and within m/z tolerance of 0.05 were included.

The EnrichNet API is limited to input of 1000 genes or proteins. Therefore, we restricted the analysis to all identified proteins with minimum ProteinProphet-estimated probability of presence of 1. Where they could be specified, i.e. in GeneTrail2 and g:Profiler, a minimum enriched set size of 5 and a maximum set size of 1000 was used. The workflows were run on the same day (Nov 8, 2017) with versions 1.1 (EnrichNet), 1.5 (GeneTrail2) and 0.6.1 (g:Profiler R package) of the enrichment analysis tools. GeneTrail2 used release 31 (20150901) of the ConsensusPathDB-human database, including KEGG. The g:Profiler used annotations from the KEGG FTP Release June 19, 2017.

The three workflows could all successfully analyze the eight test datasets. The results from the three different enrichment analyses are however not identical. As the data were collected in a platelet activation experiment, we would (somewhat naïvely) expect ‘platelet activation’ [KEGG:04611/GO:0030168] to be the most enriched pathway or annotation. However, only g:Profiler returns ‘platelet activation’ as the most enriched term. In GeneTrail2, it appears in the eighth spot. EnrichNet does not find this annotation to be enriched at all. The top 6 (and 19 of the top 25) enriched annotations/pathways found by EnrichNet (based on Comet search results) were also among the 25 most significantly enriched in at least one of GeneTrail2 and g:Profiler. The top 25 results for the three enrichment analysis tools are found in Supplementary Table S2.

**Use case no. 3.** Two workflows were selected from the PROPHETS proposals having tool combinations known to work together. To evaluate the workflows, we used a complex dataset containing a synthetic mixture of modified peptides (Chalkley *et al.*, 2013), available at ftp://iprg_public:ABRFftp.peptideatlas.org/2012/distro, providing ground truth allowing comparison of workflows with respect to accuracy of FDR estimation. The peptide spectra were searched against the given database.

*Workflow 1:* Converted mass spectra files were processed by the SearchGUI package, which generates a decoy database from the given combined database. Search parameters were set to precursor ion tolerance 50 ppm, fragment ion tolerance 0.1 Dalton, fixed modification was carbamidomethylation, variable modifications were oxidation of methionine, phosphorylation of serine, threonine and tyrosine, mono- and dimethylation of arginine and lysine, trimethylation and acetylation of lysine and sulfonation of tyrosine. Moreover, a maximum of three missed cleavages was defined. The database search engine was set to MS-generating function (GF)+ (Kim and Pevzner, 2014).

The MS-GF+ engine produced results were processed by PeptideShaker and written into the tab-separated values (TSV) format. Only modified peptides with 90% confidence and confident/doubtful [D-score (Vaudel *et al.*, 2013) and phosphoRS score (Taus *et al.*, 2011)] localizations were considered.

*Workflow 2:* Comet database search was carried out with the same search parameters as above. Peptide FDRs were calculated using PeptideProphet (command xinteract), where default parameters were used. False localization rates were estimated by PTMProphet and pep XMLfiles were converted to the TSV format by a script from the protk toolkit (https://github.com/iracooke/protk). The data were filtered for modified peptides, InterProphet score > 0.9 and a minimal PTMProphet score of 0.9 for PTM localization.The results from the two workflows and the list of synthetic peptides from the original paper were compared by an R script. pepXML file from Workflow 2 was imported into R using the XML library.

**Fig. 5.**
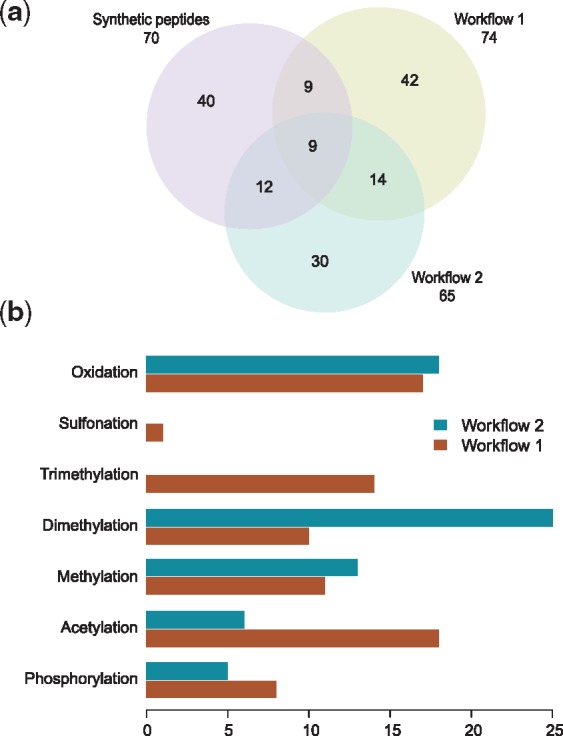
Comparison of the two workflow results in use case no. 3. (**a)** Venn diagram of identified modified peptides in both workflows and the synthetic peptides that have been added to the biological sample. (**b)** Different identification efficiency with respect to PTM types

Given the large number of variable modifications, the database search engines were forced to carry out a computationally expensive database search. In consequence, confidence estimation of peptide database search and PTM localization was driven towards their limits. Hence, we expected the results of each workflow to be biased towards e.g. certain peptide properties. Figure 5 shows exactly these trends demonstrating small overlap between the workflows as well as with the synthetic peptides that were added to the sample. We also compared how well the two workflows identified modification types. Phosphorylations and acetylations were increasingly detected in Workflow 1 while methylations and especially dimethylations gave more hits in Workflow 2. Sulfonations and trimethylations could not be distinguished from phosphorylations and acetylations when using PTMProphet.

**Use case no. 4.** To compare the output from the two iTRAQ tools proposed by PROPHETS, two workflows were generated for Libra and isobar respectively. To compare the tools and workflows, we used Orbitrap Velos FTMS datasets previously collected (Latosinska *et al.*, 2015) for a comparison of iTRAQ to label-free quantification. The raw data is available at ProteomeXchange PXD002170.

*Workflow 1:* As in use case no. 3, the data were processed by the command line tools of the SearchGUI package. Search parameters were set to precursor ion tolerance 10 ppm, fragment ion tolerance 0.05 Da, fixed modification were carbamidomethylation and iTRAQ of lysine and N-termini, variable modifications were oxidation of methionine and iTRAQ of tyrosine. Moreover, a maximum of one missed cleavages was defined. The database search engine was set to MS-GF+ and we used the Swiss-Prot human complete proteome as database (January 2017, 20 202 sequences). PeptideShaker processed the resulting files and the validated peptides were imported into R using the isobar library. Only peptides with above 80% confidence were considered.

*Workflow 2:* msconvert converted files were submitted to Comet database search with the same search parameters as above. Peptide FDRs were calculated using PeptideProphet (command xinteract) with default parameters. Quantified protein values were calculated by Libra. Subsequent comparison of expression profiles was carried out in R.

**Fig. 6.**
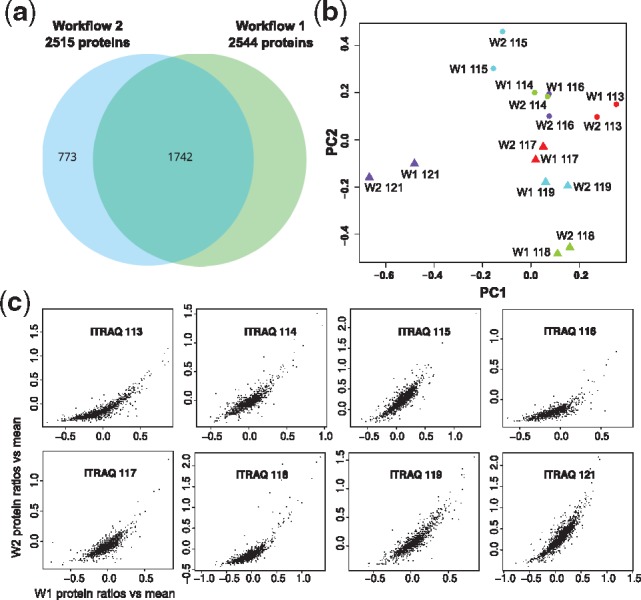
Results of use case no. 4. (**a)** Protein identification and quantification lead to an overlap of 1742 commonly quantified proteins in both workflows. (**b)** Identical samples (same color) become different for the two workflows when mapped to the first two principal components. However, they still remain sufficiently similar to distinguish biological replicates or non-muscle invasive from muscle-invasive bladder cancer. (**c)** Direct comparison of protein expression shows strong distortion at low ratios versus the mean where Workflow 2 provides much higher values. This observation can be confirmed for the other iTRAQ channels

We present the final result of the workflows by providing relative protein expression values for samples of non-muscle invasive and muscle invasive bladder cancer of four patients each. Operations like peptide database search and protein quantification require multiple algorithms for optimal performance. The methods used differ between the two workflows and we expected the results to be different to a certain degree. Figure 6 shows the results of the quantification. Both workflows quantify around 2500 proteins with an overlap of about two thirds. The 1742 commonly quantified proteins exhibit similar expression values with respect to expression changes between different biological replicates or cancer types. Direct comparison of protein expression shows agreement over a wide range but low expression values. Such a discrepancy most likely leads to diverging groups of differentially regulated proteins, and therefore has the potential to yield different biological interpretations.

## 4 Discussion

We have shown that the specification of operations, data types and formats enables the identification of compatible tools and composition of a set of tentatively viable workflows as permutations of a data analysis plan. Compatibility inferred from tool annotation does not, however, guarantee interoperability or a well-working workflow; some tools are optimized for particular datasets and may not work well, or even at all, on other data of the same type and format. To assess tool interoperability, it is necessary to benchmark them in multiple workflows. We demonstrated how automatic workflow composition suggests executable ensembles of tool combinations for both specific and general tasks, providing a framework to evaluate interoperability and identify incompatibilities.

For all use cases, we found at least slightly different results when comparing the different workflows. Our assessment of reproducibility tested the robustness of data (do different experimental measurements lead to the same end results?) and analysis (do different tool combinations give similar end results?). For use cases 2–4, we found quite significant differences between the workflow results; yielding different sets of pathways (use case 2), bias towards different PTM types (use case 3) and different quantification of low ratios (use case 4). This strongly suggests that as many workflows as feasible should be benchmarked on ‘ground truth’ data (e.g. from https://abrf.org/research-group/proteome-informatics-research-group-iprg) to identify optimal tool combinations. With the approach presented here, it will now be possible to compare many new pipeline instances to commonly used workflows on the basis of benchmarking datasets and therefore identify best-suited alternatives for specific groups of operations as well as for data types (e.g. different MS instruments). Benchmarking however might suffer from distinct performance when considering different data types such as different experimental setups or different biological sources. Thus still big community efforts will be required to create sets of different ground truth data that allow for generalized conclusions.

We showed only the results of a few workflow instances, but saw that one can, for example, easily obtain thousands of candidate workflows in use case no. 1 even from our small collection of annotated tools. The combinatorial explosion can be contained somewhat by employing more constraints in the workflow synthesis, describing the intended solution better and thus making the solution space smaller. In the case of too many proposed workflows, filtering by context knowledge like known tool compatibility, simultaneous availability in dedicated workflow management systems (e.g. GALAXY or KNIME) and software rankings will decrease their number. Alternatively, fewer workflows will be obtained by restricting the workflow synthesis to a smaller number of well-known tools. For simplicity, our study was restricted to single input/output descriptors, but in reality many tools have multiple input or output data types and support multiple file formats. If such annotations or a larger tool collection were considered, more flexible ways to process the candidate workflows would be needed. Instead of just ranking the solutions by length, the framework could, e.g. apply estimated computational efficiency, or information from bio.tools such as license, supported platform, accessibility and cost, to limit the search space, or even provide an interactive interface that lets a user browse through possible solutions much like an online route planner.

Regarding maintenance, workflows constructed from multiple, independently developed tools may appear fragile, as the failure (or unavailability) of any single link in the chain will break the workflow. However, since such tools are performing a single, well-defined task, they are easier to replace. Most operations in proteomics data analysis at least, are well served by multiple tools in the public domain. It is therefore crucial to have tools formally annotated with respect to input/output formats and the type of operations performed.

All the tools and annotation terms required for this work have been registered in bio.tools and EDAM, respectively. At the outset, bio.tools included few tools for proteomics analysis and EDAM only a few relevant concepts. Creating the required EDAM concepts and tool annotations involved expert understanding of the field, and knowledge of specific tools gained from reading documentation or publications, with cycles of curation to ensure semantic consistency. In some cases, unexpected synthesis results indicated imprecise or erroneous domain modeling, and led to revisions of the ontology or individual tool annotations. Thus, a substantial manual effort was required for tool annotation and ontology development. Nonetheless, the groundwork is laid for future studies in proteomics that may also serve as an exemplar for other domains. The bio.tools data and EDAM are freely accessible for community additions, extension and re-use, and with progressive expert curation can provide a rich source of information for generating domain models for workflow synthesis applications. For example, in future publications we are going to describe the process and best practices of ontological modelling in EDAM and semantic annotation in bio.tools in general and in particular target a more comprehensive coverage of the prevalent proteomics analysis tools and concepts. We anticipate, for scenarios similar to our use cases, to generate workflows from a much larger set of tools, or eventually even the complete bio.tools corpus of currently >10 000 software tools and databases. Workflow registration, and annotation of underlying tool operations and their supported data formats, are core goals of bio.tools which will support future workflow composition efforts.

In this article, we have demonstrated a proof of concept. Much further work is needed to provide an implementation suitable for the non-expert bench user, and many challenges stand in the way of this vision. In summary, this includes the modelling of complex tools with multiple inputs and outputs, flexible ways to rank and filter prospective workflows, the algorithmic complexity of synthesis algorithms, specification and synthesis of non-linear workflows (i.e. workflows with parallelism, loops or conditional branchings), provision of reliable benchmarking datasets for different analyses, and the automated implementation of workflow solutions, to name a few. We envision to work together with the developer of workflow management systems, to integrate our method there to provide additional workflow composition support to their users. For example, if running the synthesis from within, say, a Galaxy server, then it could be made aware of what software tools are available on that server. Workflow generation could then be restricted to already installed software, or components already available on the server could be prioritized over those that are not. Furthermore, the synthesized workflows could be exported in exchange formats like the Common Workflow Lanugage (Amstutz *et al.*, 2016) and provided along with containerized tool packages to facilitate their execution on various platforms.

In summary, we have taken another small step towards plug-and-play workflow composition, where researchers only need to specify the overall setup of their bioinformatics pipeline to automatically receive directly executable and benchmarked software solutions. We believe that the field is ripe for further work, and we welcome collaborations in all areas.

## Supplementary Material

Supplementary Data
