# Supplementary figures and images for: Automated workflow composition in mass spectrometry-based proteomics
Source: Bioinformatics. 2018 Jul 24;35(4):656–64. doi: 10.1093/bioinformatics/bty646 (PMC6378944; doi:10.1093/bioinformatics/bty646)

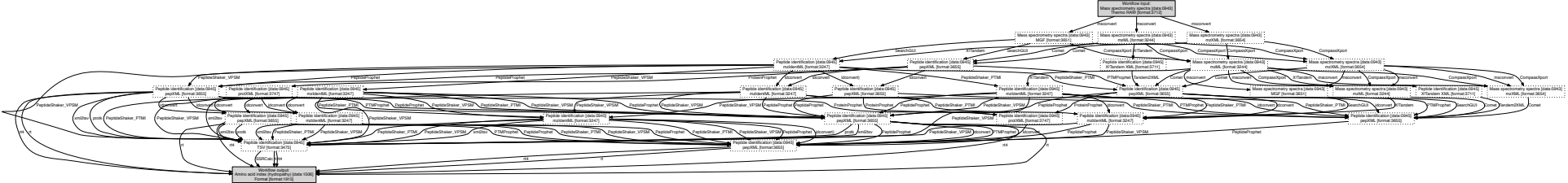

Supplement: Supplementary Data [file bty646_supp.zip › bty646-suppl_data/bty646_Supplementary_Figure_3.pdf]

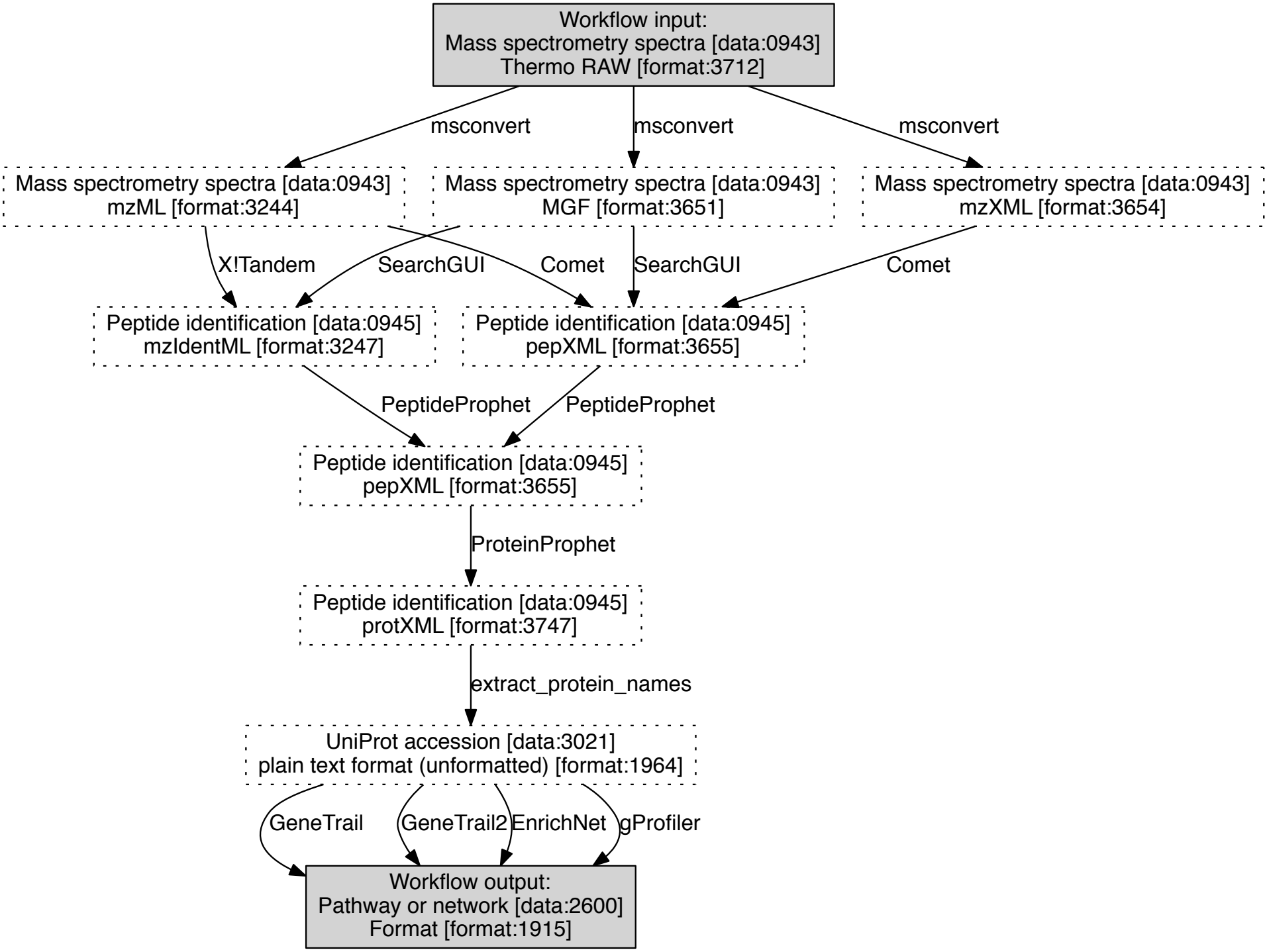

Supplement: Supplementary Data [file bty646_supp.zip › bty646-suppl_data/bty646_Supplementary_Figure_4.pdf]

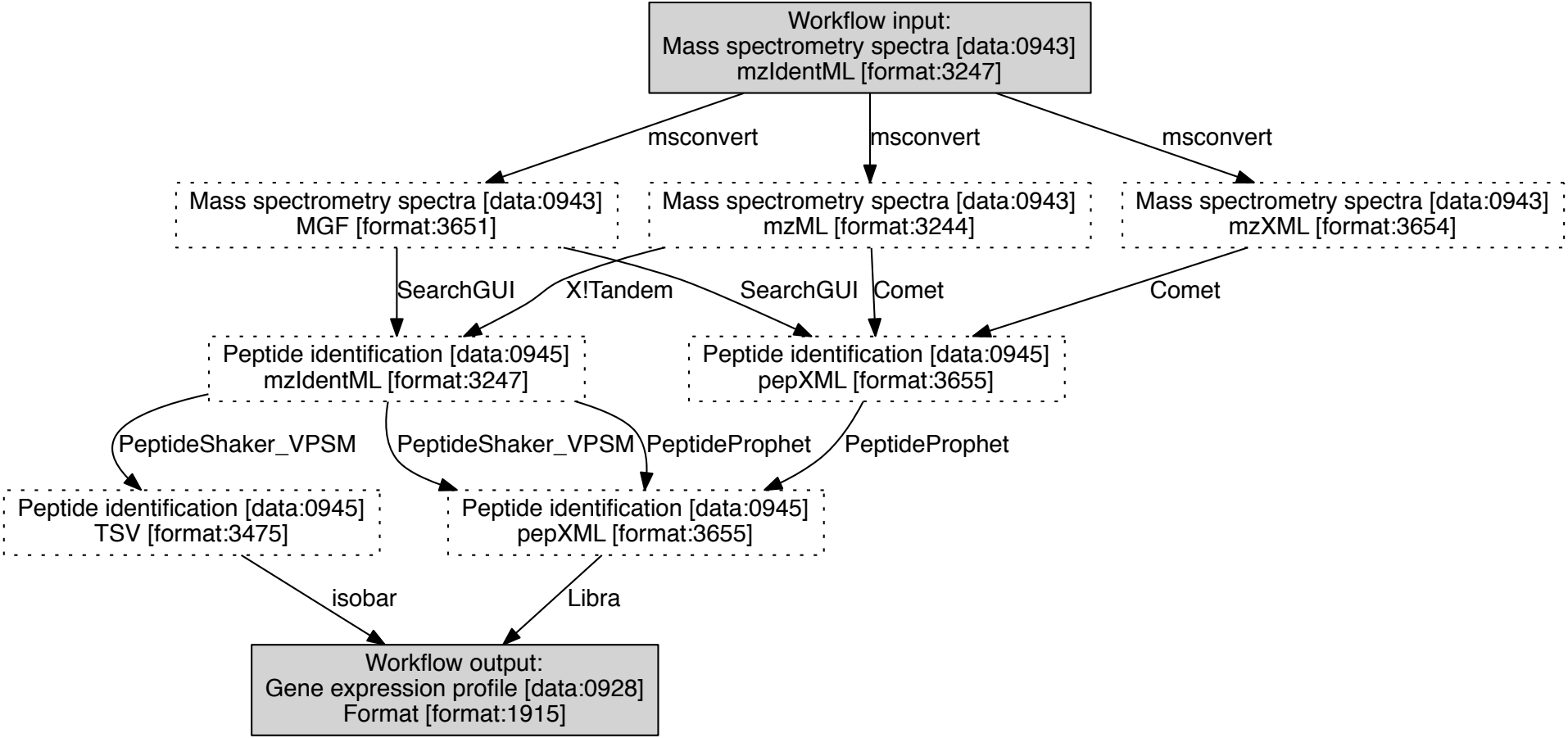

Supplement: Supplementary Data [file bty646_supp.zip › bty646-suppl_data/bty646_Supplementary_Figure_6.pdf]

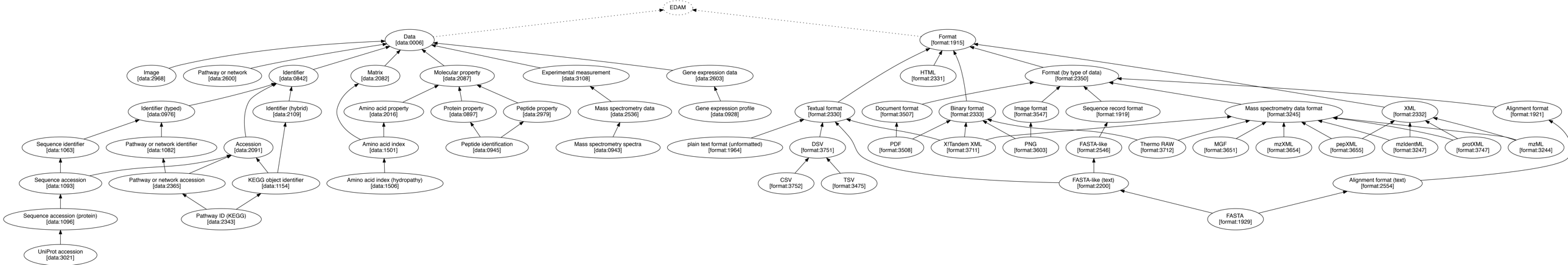

Supplement: Supplementary Data [file bty646_supp.zip › bty646-suppl_data/bty646_Supplementary_Figure_1.pdf]

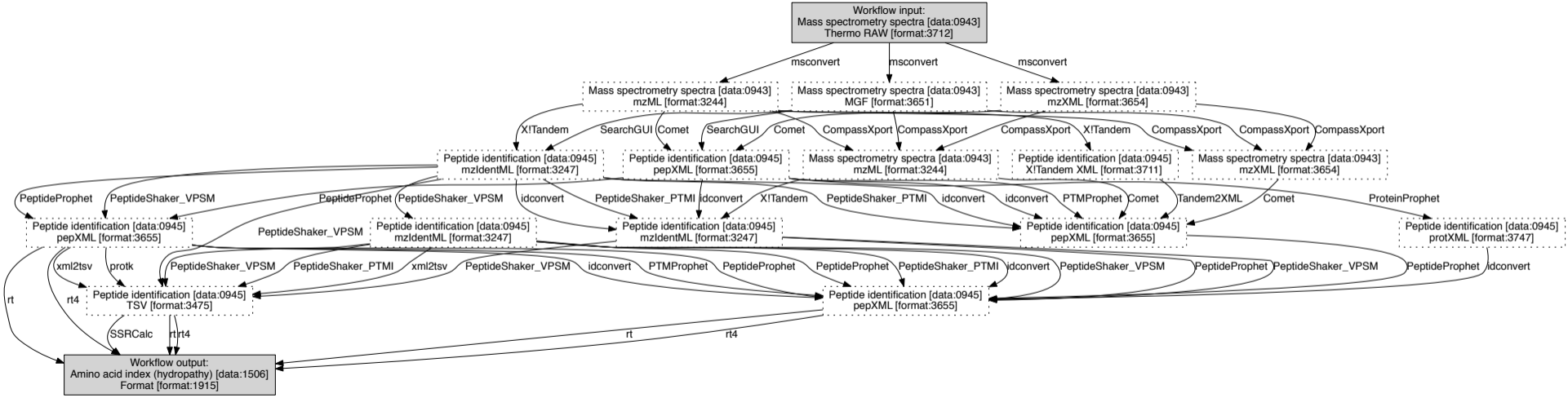

Supplement: Supplementary Data [file bty646_supp.zip › bty646-suppl_data/bty646_Supplementary_Figure_2.pdf]

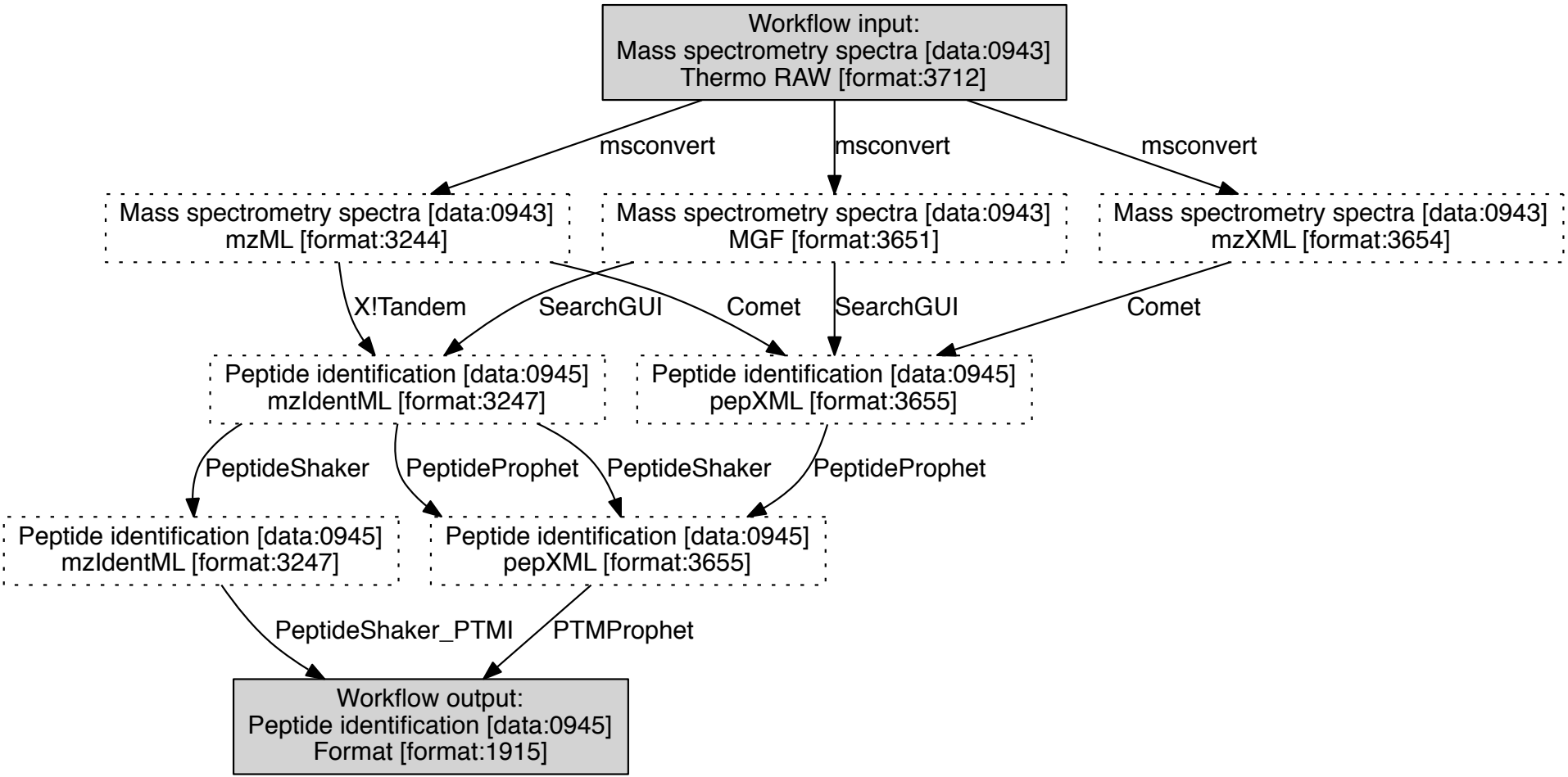

Supplement: Supplementary Data [file bty646_supp.zip › bty646-suppl_data/bty646_Supplementary_Figure_5.pdf]
